# Supplementary material for: Enhancing Coherence with a Clock Transition and Dynamical Decoupling in the Cr7Mn Molecular Nanomagnet
Source: ACS Nanosci Au. 2026 Jan 10;6(2):324–38. doi: 10.1021/acsnanoscienceau.5c00192 (PMC13087941; doi:10.1021/acsnanoscienceau.5c00192)
Supplement: Supplementary file 1 [file ng5c00192_si_001.pdf]

Supporting Information for Enhancing Coherence with a Clock Transition and Dynamical Decoupling in the Cr_7Mn Molecular Nanomagnet

Guanchu Chen,[1,2] Brendan C. Sheehan,[1,2,*] Ilija Nikolov,[1,†] James W. Logan,[1,*] Charles A. Collett,[1,‡] Gajadhar Joshi,[1,§] Grigore A. Timco,[3] Jillian E. Denhardt,[4,¶] Kevin R. Kittilstved,[4,**] Richard E. P. Winpenny,[3] and Jonathan R. Friedman[1,2,††]

[1]*Department of Physics and Astronomy, Amherst College, Amherst, MA 01002, USA*

[2]*Department of Physics, University of Massachusetts Amherst, Amherst, MA 01003, USA*

[3]*Department of Chemistry, The University of Manchester, Manchester M13 9PL, UK*

[4]*Department of Chemistry, University of Massachusetts, Amherst, MA 01003, USA*

(Dated: July 18, 2025)

S-I. INHOMOGENEOUS BROADENING

The inhomogeneous broadening can be seen in the broad response of ESR echo signal over a wide range of frequencies. As shown in Fig. S1, at zero field we see significant echo signal across a frequency range of $\sim$2.5 GHz, with 3 different resonators — the frequency of each resonator is tuned over the range indicated by a single color through use of a dielectric. At zero field, the transitions frequency is determined exclusively by the transverse anisotropy: $\epsilon = 2E$; thus, the observations provide a direct indication of the inhomogeneity in E. It is

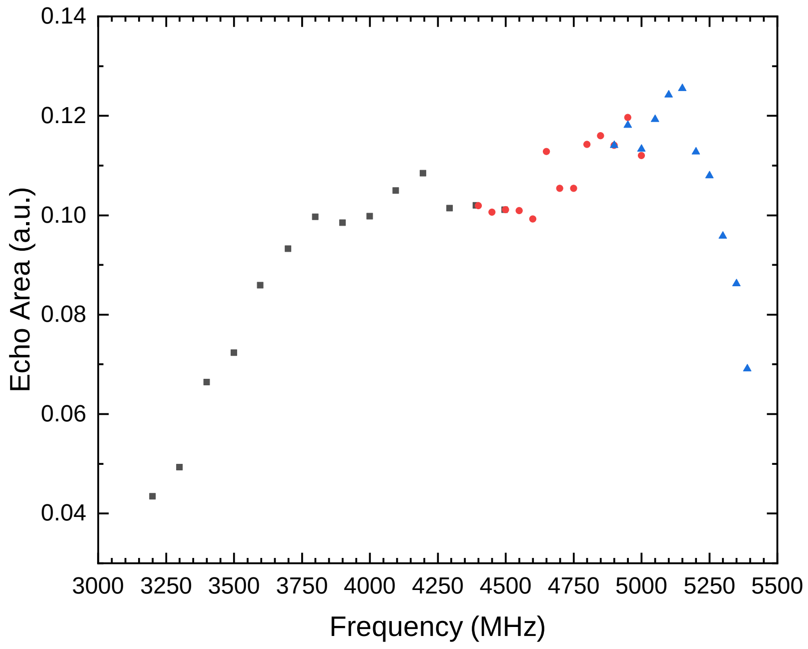

FIG. S1. Zero-field frequency response of **2** at the clock transition. The data is from 1% toluene solution sample at 1.8 K with three different resonators (marked with colors; to stitch together data from different resonators, a shared frequency in the overlap region is used to normalize the echo area), indicating a very broad frequency response. Since this is a zero-field signal, we can infer a broad inhomogeneity in the anisotropy parameter E.

* Current address: Department of Physics and Astronomy, Dartmouth College, Hanover, NH 03755, USA
† Current address: Department of Physics, Brown University, Providence, RI 02912, USA
‡ Current address: Department of Physics, Hamilton College, Clinton, NY 13323, USA
§ Current address: Center for Integrated Nanotechnologies, Sandia National Laboratories, Albuquerque, NM 87123, USA
¶ Current address: University of Hawai‘i at Mānoa, Honolulu, HI 96822, USA
** Current address: Department of Chemistry, Washington State University, Pullman, 99164-4630, USA
†† jrfriedman@amherst.edu

worth noting that we have seen echo beyond this range; the data shown is from a particular experiment with controlled frequency response.

S-II. HOLE BURNING

Despite the broad inhomogeneity, at any given drive frequency, we are only exciting a small sub-ensemble of the spins. This is demonstrated with a hole-burning experiment, illustrated in Fig. S2(a). The sequence contains three pulses: a hole-burning pulse is followed after a time τ' by a traditional Hahn echo sequence (π/2-τ-π-τ-echo), where τ is the delay time between Hahn pulses. The hole-burning pulse is detuned from the working ESR frequency by some detuning frequency Δf, which becomes the independent variable of the experiment. The hole-burning pulse has a duration of 2 µs so that its linewidth is small compared to those of Hahn sequence pulses. The amplitude of the hole-burning pulse is kept small to prevent possible saturation effects. By sweeping Δf a hole is measured (an example is shown in Fig. S2(b)) and fit with a Voigt function. By changing time τ', we are able to map the energy diffusion over time. From Fig. S2(c), we see that the full width at half maximum of the hole changes from ~ 1.5 MHz to ~ 2 MHz over 50 µs of evolution. Our typical pulsed experiments were done within the time scale of tens of microseconds, meaning we only probed the resonant sub-ensemble of the system, before any significant spectral diffusion had occurred.

S-III. INVERSION RECOVERY

Fig. S3 shows an example of an inversion recovery experiment to measure T_1. Due to the instrumental limitations, it doesn't fully recover within the accessible parameters. The data shows a double exponential decay with two time constant 63(6) µs and 1140(35) µs, well above T_2 and all relevant time scales in our experiments, showing that the longitudinal relaxation is not the limiting factor in our system.

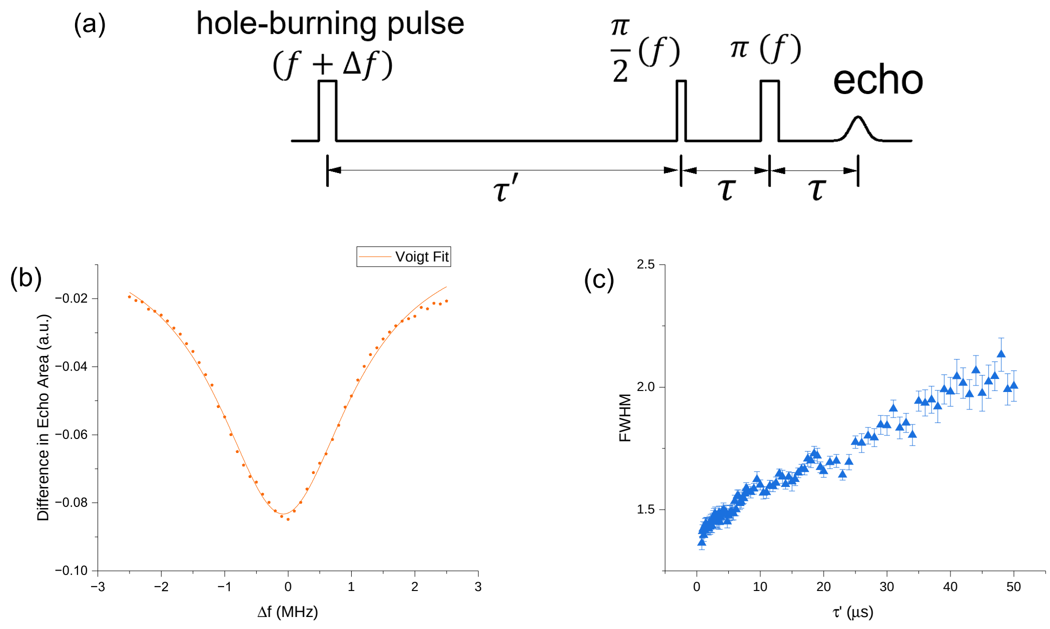

FIG. S2. Hole Burning Experiment. a) Pulse diagram of the hole-burning sequence. A delay of τ' follows the hole-burning pulse after which echo is detected with a Hahn sequence. b) Example of a burned hole. The hole-burning pulse is “soft”, having a long width of 2 µs. Data is measured with 10% dilution **2** toluene solution at 1.8 K and 5625 MHz. c) Evolution of FWHM of the burned hole. The diffusion happens slowly compared to the typical timescale of our pulsed experiments, i.e. T_2: after 50 µs, the hole width increases from $\sim$1.5 MHz to $\sim$2 MHz.

S-IV. COMPARISON OF SAMPLE VARIANTS, DILUTIONS AND COOLING TECHNIQUES

Fig. S4 shows a comparison of echo signals from different Cr_7Mn sample variants, dilutions and cooling techniques. While signal strength naturally varies — largely due to concentration and also due to variations in the measurement circuit and other experimental conditions — we expect the coherence time T_2 to be more governed by intrinsic physical properties, and to exhibit more consistent and interpretable trends. The figure compares T_2 values for several samples, measured at a variety of frequencies. The most naive expectation is that T_2 should be enhanced by further dilution. However, we see very little change as concentration varies between 0.1% and 10%. When using the CPMG sequence — Fig. S5 — we do see some hint of a dependence of T_2 on concentration, but it is hard to draw strong conclusions from the data.

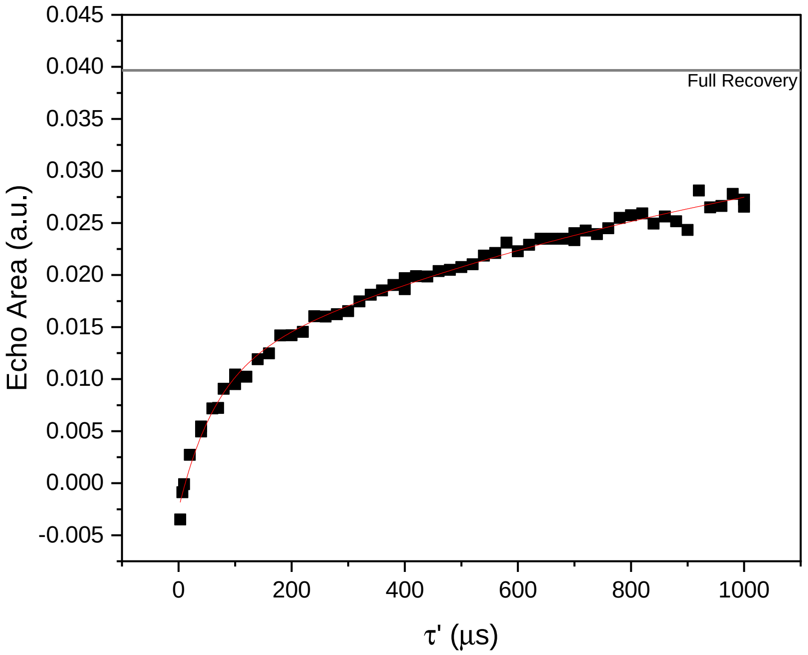

FIG. S3. Inversion recovery experiment measuring T_1. Similar to what we had in hole-burning experiment, with hole-burning pulse replaced by a π pulse, we can measure the longitudinal relaxation time T_1 by varying τ'. The data is fitted with a double exponential decay with two time constants 63(6) μs and 1140(35) μs. The fully recovered level is determined by performing the experiment without the π preparation pulse. Data is measured with 1% dilution **2** toluene solution at 1.8 K and 5350 MHz.

The measured T_2 value also does not seem to depend significantly on the solvent: liquid solutions using toluene or deuterated toluene solvents were studied as well as solid solutions by co-crystallizing Cr_7Mn with Ga_7Zn; all show rather similar behavior. Variation of cation (comparing **1** with **2**) was also not found to have significant effect on the coherence. This indicates that the most pronounced noise contributing to T_2 originates from within the molecule itself.

S-V. GAUSSIAN INTEGRAL

Upon averaging over the noise, we find that all noise contributions enter through factors of $\cos\phi$, where ϕ represents the accumulated noise-induced phase. Owing to the central limit

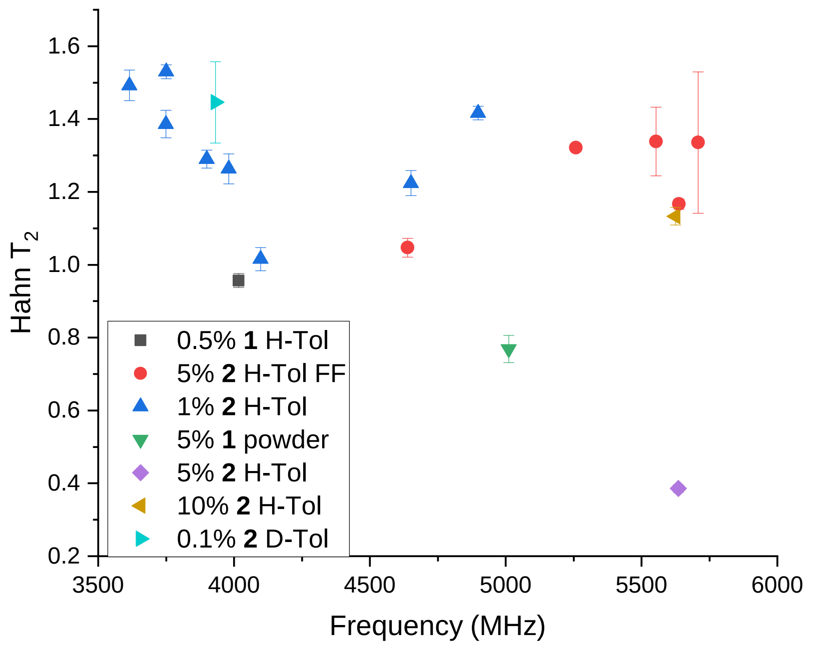

FIG. S4. Frequency dependency of Hahn T_2 at the zero-field clock transition from different sample variants, dilutions, solvent and cooling techniques. D-Tol indicates deuterated toluene, FF indicates a sample that was flash frozen, and powder refers to a solid solution sample of Cr_7Mn in Ga_7Zn.

theorem, each accumulated phase is treated as a zero-mean stationary Gaussian random variable. For a ϕ with standard deviation σ, i.e., $\phi \sim \mathcal{N}(0, \sigma^2)$, then

$$\begin{aligned}\mathbb{E}\left[\cos\phi\right] = \mathbb{E}\left[e^{i\phi}\right] &= \int \mathrm{d}\phi \, \frac{e^{i\phi}}{\sigma\sqrt{2\pi}} e^{-\phi^2/(2\sigma^2)} \\ &= \exp\left(-\frac{1}{2}\sigma^2\right) \int \mathrm{d}\phi \, \frac{1}{\sigma\sqrt{2\pi}} \exp\left(-\frac{(\phi - i\sigma^2)^2}{2\sigma^2}\right) \\ &= \exp\left(-\frac{1}{2}\sigma^2\right) \equiv \exp\left(-\frac{1}{2}\mathbb{E}[\phi^2]\right)\end{aligned}$$

S-VI. NOISE SPECTRUM CONVERSION COEFFICIENTS

We derive the conversion coefficients from the noise on $\boldsymbol{B}$ to noise on ϵ by solving the Hamiltonian and then expanding it around the external field $\boldsymbol{B}_0$. In Einstein notation,

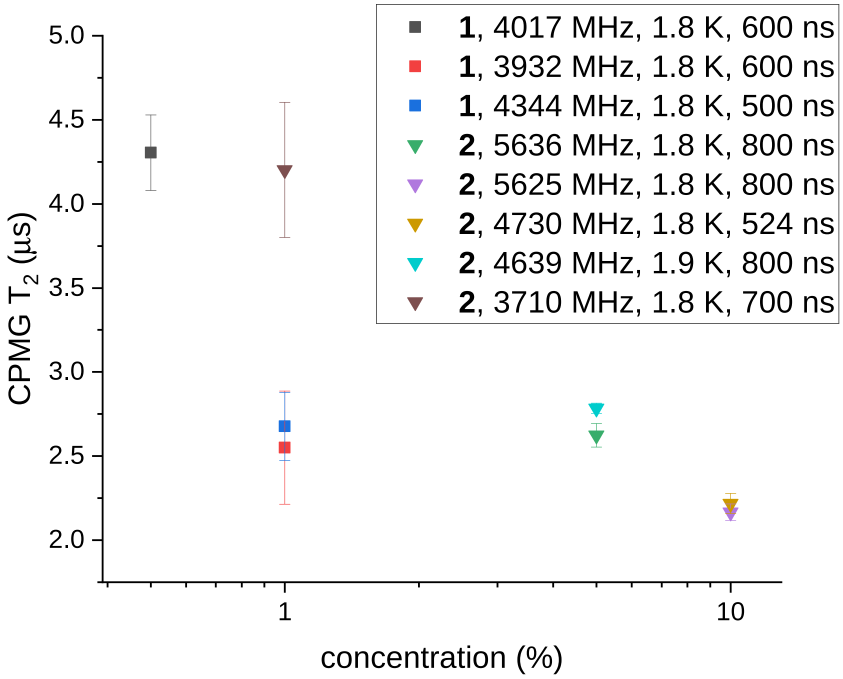

FIG. S5. CPMG T_2 dependence on dilution. The data is from various samples, as noted in legend, with frequencies, temperature, and τ of the CPMG sequences. The data appear to indicate longer T_2 with lower dilution, but the scatter makes it hard to infer strong conclusions.

denoting $\epsilon_i \equiv \frac{\partial \epsilon}{\partial B^i}$ and $\epsilon_{ij} \equiv \frac{\partial^2 \epsilon}{\partial B^i \partial B^j}$, a Taylor series expansion yields

$$\epsilon = \epsilon_0 + \underbrace{B^i \epsilon_i}_{2nd} + \underbrace{B^i B^j \epsilon_{ij}}_{3rd} + \underbrace{\frac{\partial \epsilon}{\partial E} \delta E}_{4th}, \tag{S1}$$

where $B^i \equiv \int r^2 \mathrm{d}r \mathrm{d}\Omega b^i(\boldsymbol{r})$ is the collective induced field of the proton bath at the origin (location of the electronic spin), and δE is the dynamical deviation of anisotropy parameter E. The field $b^i(\boldsymbol{r})$ is defined below, cf. Eq. S10. It is easy to see there is no cross-correlation between terms (i.e., 2nd, 3rd, and 4th terms). Thus, to get the temporal autocorrelation of $\delta\epsilon = \epsilon - \mathbb{E}[\epsilon]$, one can calculate the contribution of each term separately. For the second term in Eq. S1, we can get the contribution to $\mathbb{E}[\delta\epsilon(t_1)\delta\epsilon(t_2)]$:

$$\mathrm{corr}_2 \equiv \mathbb{E}[\delta\epsilon(t_1)\delta\epsilon(t_2)] = \epsilon_i \epsilon_j \, \mathbb{E}[B^i(t_1) B^j(t_2)]. \tag{S2}$$

We assume the field coming from protons at different locations is uncorrelated, so

$$\mathbb{E}[B^i(t_1) B^j(t_2)] = \int r^2 \mathrm{d}r \mathrm{d}\Omega \, \mathbb{E}[b^i(\boldsymbol{r}, t_1) b^j(\boldsymbol{r}, t_2)] \tag{S3}$$

As shown in the next section, $\mathbb{E}[b^i(\boldsymbol{r},t_1)b^j(\boldsymbol{r},t_2)] = R_2(r)g^{i,j}(\theta,\phi)\cos(\omega_{L,0}T)\exp(-\frac{1}{2}\sigma|T|)$ with $T \equiv t_2 - t_1$, given our assumption that the noise arises from Larmor precession at frequency $\omega_{L,0}$. Then

$$\mathrm{corr}_2 = \cos(\omega_{L,0}T)e^{-\frac{1}{2}\sigma|T|}\int R_2(r)r^2\mathrm{d}r\int \mathrm{d}\Omega g^{i,j}(\theta,\phi)\epsilon_i\epsilon_j \tag{S4}$$

$$\equiv \alpha C_1\cos(\omega_{L,0}T)e^{-\frac{1}{2}\sigma|T|}, \tag{S5}$$

defining $\alpha \equiv \int R_2(r)r^2\mathrm{d}r$ a fitting parameter reflecting the radial distribution in protons, and $C_1 \equiv \epsilon_i\epsilon_j\int \mathrm{d}\Omega g^{i,j}(\theta,\phi)$, which can be calculated assuming an isotropic distribution of protons.

The same process can be carried out for the 3rd and 4th terms to obtain

$$\mathrm{corr}_3 = \cos(2\omega_{L,0}T)e^{-2\sigma|T|}\int R_4(r)r^2\mathrm{d}r\int \mathrm{d}\Omega g_2^{ijkl}(\theta,\phi)\epsilon_{ij}\epsilon_{kl} \tag{S6}$$

$$\equiv \beta C_2\cos(2\omega_{L,0}T)e^{-2\sigma|T|}, \tag{S7}$$

$$\mathrm{corr}_4 = \left(\frac{\partial\epsilon}{\partial E}\right)^2\mathbb{E}[\delta E(t_1)\delta E(t_2)] \tag{S8}$$

$$\equiv C_3\,\mathbb{E}[\delta E(t_1)\delta E(t_2)], \tag{S9}$$

in which $\beta \equiv \int R_4(r)r^2\mathrm{d}r$, $C_2 \equiv \epsilon_{ij}\epsilon_{kl}\int \mathrm{d}\Omega g_2^{ijkl}(\theta,\phi)$, $C_3 \equiv \left(\frac{\partial\epsilon}{\partial E}\right)^2$, $\mathbb{E}[\delta E(t_1)\delta E(t_2)] = \int\frac{\mathrm{d}\omega}{2\pi}e^{-i\omega t}\gamma\cdot\frac{1}{\omega}$, following the *ad hoc* assumption of a $1/f$ spectrum for the noise in E.

S-VII. NOISE ON LARMOR FREQUENCY

We treat each nuclear spin classically, as a magnetic dipole moment $\boldsymbol{\mu}$ at position $\boldsymbol{r}$. The induced magnetic field at the electronic spin located at the origin is

$$b^i(\boldsymbol{r}) = \frac{\mu_0\mu}{4\pi r^3}\left(3\hat{r}^i\hat{r}_m\hat{\mu}^m - \hat{\mu}^i\right) \tag{S10}$$

$$= \frac{\mu_0\mu}{4\pi r^3}\left(3\hat{r}^i\hat{r}_m - \delta^i_m\right)\hat{\mu}^m \tag{S11}$$

Then, for corr_2, one obtains

$$\mathbb{E}[b^i(\boldsymbol{r},t_1)b^j(\boldsymbol{r},t_2)] = R_2(r)\left(3\hat{r}^i\hat{r}_m - \delta^i_m\right)\left(3\hat{r}^j\hat{r}_n - \delta^j_n\right)\mathbb{E}[\hat{\mu}^m(t_1)\hat{\mu}^n(t_2)] \tag{S12}$$

$$\equiv R_2(r)\Theta^{ij}_{mn}(\theta,\phi)\,\mathbb{E}[\hat{\mu}^m(t_1)\hat{\mu}^n(t_2)], \tag{S13}$$

where $R_2(r) = \left(\frac{\mu_0\mu}{4\pi r^3}\right)^2$ and $\Theta^{ij}_{mn}(\theta,\phi) = (3\hat{r}^i\hat{r}_m - \delta^i_m)(3\hat{r}^j\hat{r}_n - \delta^j_n)$. Similarly, for corr$_3$, one finds

$$\mathbb{E}[b^i(\boldsymbol{r},t_1)b^j(\boldsymbol{r},t_1)b^k(\boldsymbol{r},t_2)b^l(\boldsymbol{r},t_2)] = R_4(r)\Theta^{ijkl}_{mnpq}(\theta,\phi)\,\mathbb{E}[\hat{\mu}^m(t_1)\hat{\mu}^n(t_1)\hat{\mu}^p(t_2)\hat{\mu}^q(t_2)] \quad \text{(S14)}$$

For convenience, we define a new frame (XYZ) with the precession axis as the Z-axis so

$$\hat{\mu}_X = \hat{\mu}_\perp \cos(\int \mathrm{d}t\omega_L + \varphi) \quad \text{(S15)}$$

$$\hat{\mu}_Y = \hat{\mu}_\perp \sin(\int \mathrm{d}t\omega_L + \varphi) \quad \text{(S16)}$$

$$\hat{\mu}_Z = \hat{\mu}_\parallel \quad \text{(S17)}$$

in which $\hat{\mu}_\perp$, $\hat{\mu}_\parallel$, and φ define the initial state at $t = 0$, which is assumed random, and $\omega_L = \omega_{L,0} + \delta\omega_L$. To illustrate how we get the damping cosine function in Eq. S4 and Eq. S6, we show how we calculate $\mathbb{E}[\hat{\mu}_X(t_1)\hat{\mu}_X(t_2)]$:

$$\mathbb{E}[\hat{\mu}_X(t_1)\hat{\mu}_X(t_2)] = \mathbb{E}[\hat{\mu}_\perp^2 \cos(\int_0^{t_1} \mathrm{d}t\omega_L + \varphi)\cos(\int_0^{t_2} \mathrm{d}t\omega_L + \varphi)] \quad \text{(S18)}$$

$$= \frac{1}{3}\,\mathbb{E}[\exp\left[i\left(\omega_{L,0}(t_2 - t_1) + \int_{t_1}^{t_2} \mathrm{d}t\delta\omega_L(t)\right)\right] + H.C.] \quad \text{(S19)}$$

$$= \frac{2}{3}\cos(\omega_0 T)\exp\left[-\frac{1}{2}\sigma|T|\right], \quad \text{(S20)}$$

obtained by first averaging over initial states (Eq. S19) ($\int \hat{\mu}_\perp^2 \sin\vartheta \mathrm{d}\vartheta = \frac{4}{3}$, $\int \exp(2i\varphi)\,\mathrm{d}\varphi = 0$) and then the noise in ω_L (Eq. S20), in which $\int_{t_1}^{t_2} \mathrm{d}t\delta\omega_L(t)$ has a Gaussian distribution of $N\left(0, \sqrt{\sigma|T|}\right)$. A similar calculation can be carried out for other terms and all terms in the new frame can be converted back to the molecule frame with a rotation matrix. All together, we can get $\mathbb{E}[\hat{\mu}^m(t_1)\hat{\mu}^n(t_2)] = G^{mn}\cos(\omega_0 T)\exp\left[-\frac{1}{2}\sigma|T|\right]$, where G^{mn} is a matrix of constants reflecting the symmetry of our system. Plugging this back to Eq. S13, we get $g^{i,j}(\theta,\phi) \equiv \Theta^{ij}_{mn}(\theta,\phi)G^{mn}$. Similarly, for corr$_3$, $\mathbb{E}[\hat{\mu}^m(t_1)\hat{\mu}^n(t_1)\hat{\mu}^p(t_2)\hat{\mu}^q(t_2)] = 2G^{mnpq}\,\mathbb{E}[\exp\left[i\left(2\omega_{L,0}(t_2 - t_1) + 2\int_{t_1}^{t_2} \mathrm{d}t\delta\omega_L(t)\right)\right] + H.C.] = G^{mnpq}\cos(2\omega_{L,0}T)\exp[-2\sigma|T|]$.

S-VIII. ADDITIONAL DATA/SIMULATIONS

Fig. S6–S9 show the data at other field not shown in the main text but used in the fitting.

S-IX. FITTING WITHOUT NOISE IN E

As presented in the main text, our noise/decoherence model includes field noise due to a nuclear spin bath as well as noise in the anisotropy parameter E, $\mathcal{S}_E \propto 1/f$. This noise in E presents a limiting factor on decoherence at the CT, where the effects of field fluctuations are largely filtered out. At zero field, where the CT occurs, the only noise sources in our model that can lead to decoherence are $\mathcal{S}_{\boldsymbol{B}_I \otimes \boldsymbol{B}_I}$ and $\mathcal{S}_E$ since $\mathcal{S}_{B_I}$ is filtered completely by the CT. This raises the question of whether the second-order field fluctuations $\mathcal{S}_{\boldsymbol{B}_I \otimes \boldsymbol{B}_I}$ could be sufficient to account for our observed decoherence at zero field. Re-running a fit of our model at zero field, forcing $\mathcal{S}_E = 0$ while allowing all other parameters to vary [1], produces the results shown in Fig. S10. The fit is far worse than what we obtain by including $\mathcal{S}_E$, as shown in Fig. 10(a) in the main text. This strongly suggests that the fluctuations in E play an essential role in decoherence at the CT and that second-order fluctuations are insufficient to account for the observed zero-field behavior.

S-X. DISCUSSION OF COHERENT MODEL ON INTERMEDIATE RANGE OF FIELD

For intermediate-range fields, there is a revival of the signal after the system has seemingly fully decohered. Fig. S11 show the Hahn echo area in the range of 50–95 Oe. This revival is in fact consistent with ESEEM oscillations. The echo area initially decays in a quasi-exponential way, but then recovers at later times, producing a signal that peaks at a time that is roughly consistent with the Larmor precession frequency. We find that this behavior

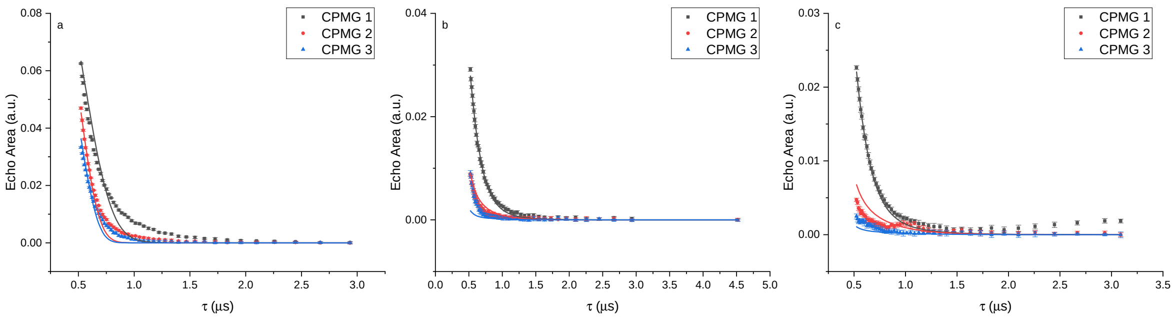

FIG. S6. Additional CPMG data (points) and fits (solid lines) at fields of (a) 25, (b) 50, and (c) 75 Oe.

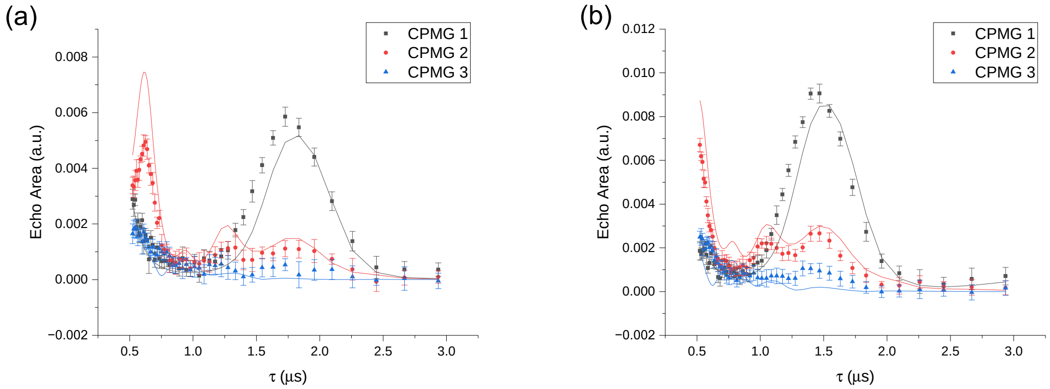

FIG. S7. Additional CPMG data (points) and fits (solid lines) at fields of (a) 125 and (b) 150 Oe.

is not well produced with our coherent-model simulations, highlighting the advantage of modeling the environment as a noise source. Figure S12 illustrates the inability of the coherent-coupling model to adequately reproduce the data in this range of fields. In the top six panels (a-f), we show experimental and simulated results for a sampling of six fields in this regime. The dashed lines represent the results of simulations without decoherence while the solid lines show the results of fitting. For the fitting, we fixed the hyperfine parameters to those found from the fitting of data in the 195 – 395 Oe field range and then refit the the data over all fields, allowing all other parameters (T_2 for each field, scaling parameter, offset) to vary. There are two important failures of the simulations here: 1) Without decoherence, the simulations show a peak at values of 2τ (e.g. $\sim$3 µs for 75 Oe) where no peak is observed in the data. This peak corresponds to oscillations at about twice the Larmor frequency, a known effect in ESEEM. 2) The fitting is unable to meaningfully reproduce the data. One important observation is that the signal size in this range of fields is approximately an order of magnitude smaller than in the higher-field range. In order to account for the small signal size as well as the rapid decay of low values of τ and the absence of the low-τ peak, the fit yields an extremely fast decay that does not account for the signal revival at longer values of τ. Attempts to force T_2 to be larger to allow for observation of the revival results in a signal amplitude that is vastly larger than the observations in this regime.

To attempt to address the issues described above, we chose to reduce the hyperfine coupling strength by more than an order of magnitude: $A_{zz} = 0.2$ MHz and $A_{z\perp} = 0$. The motivation for doing this is to eliminate the low-τ peak in the simulations. An additional

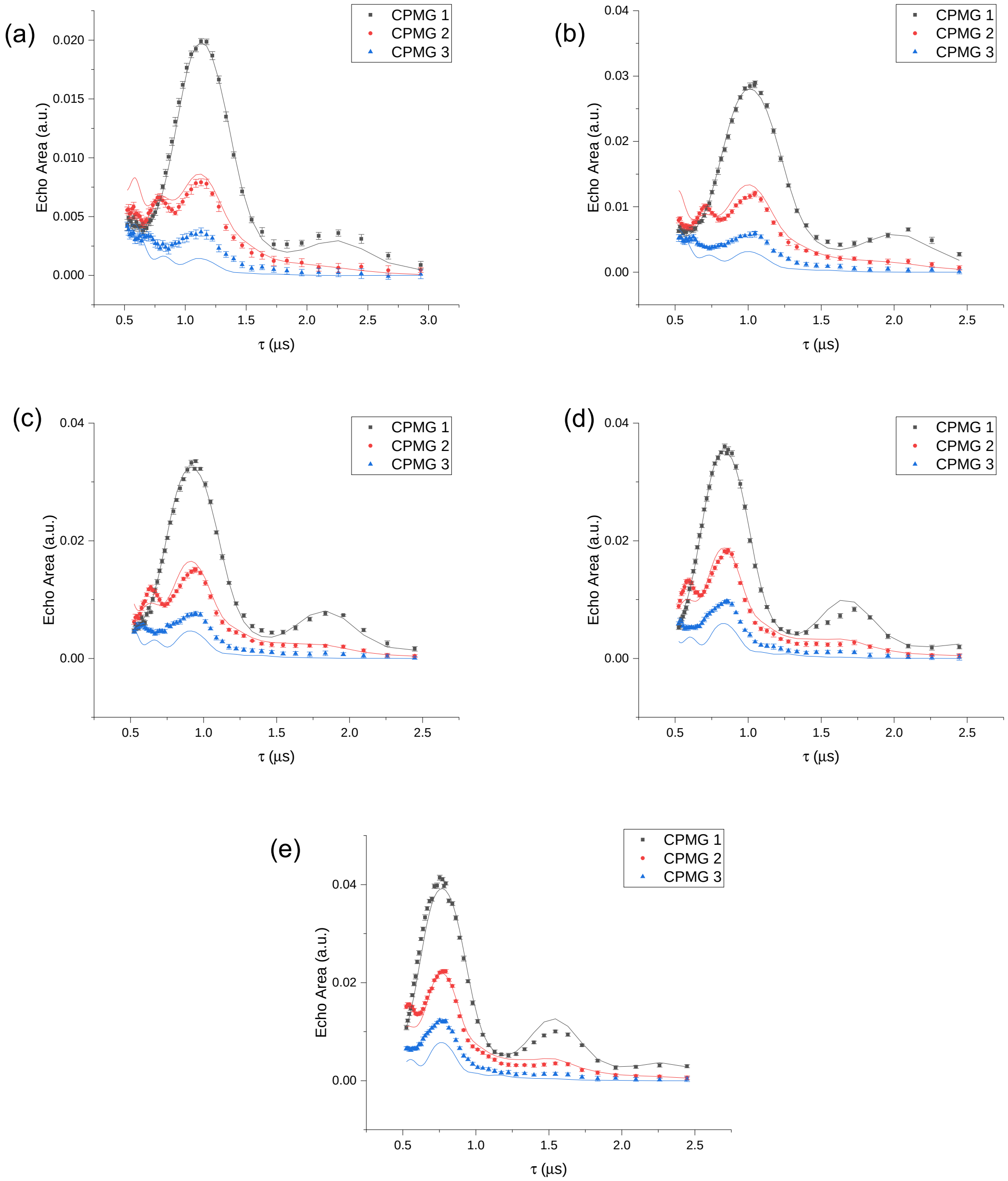

FIG. S8. Additional CPMG data (points) and fits (solid lines) at fields of (a) 200, (b) 225, (c) 250, (d) 275, and (e) 300 Oe.

consequence of reducing A_{zz} to this small value makes the amplitude of the ESEEM oscillations much smaller than the observations. To compensate for this, we allowed for coupling of the electronic spin to a large number of protons, not just one as in our previous model.

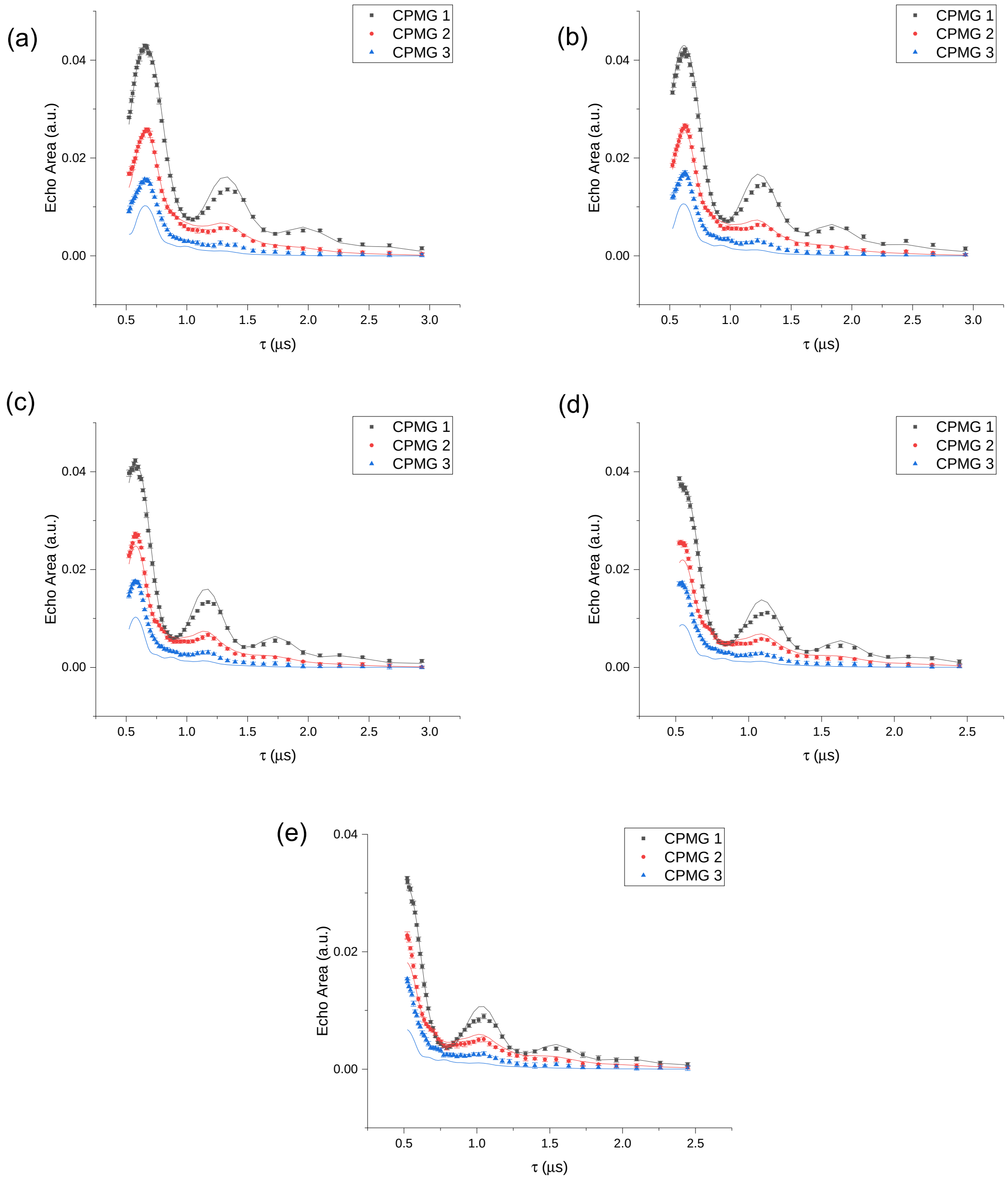

FIG. S9. Additional CPMG data (points) and fits (solid lines) at fields of (a) 350, (b) 375, (c) 400, (d) 425, and (e) 450 Oe.

In other words, we update Eq. 2 (main text) to

$$\mathcal{H} = -DS_z^2 + E(S_x^2 - S_y^2) + g_s\mu_B \boldsymbol{B}\cdot\boldsymbol{S} + \sum_i \left(A_{zz}S_zI_{i,z} - g_p\mu_p\boldsymbol{B}\cdot\boldsymbol{I}_i\right). \tag{S21}$$

For the small value of A_{zz} we use, we find that the ESEEM oscillations retain their shape as we increase the number of protons n_p in the sum and the oscillation amplitude scales

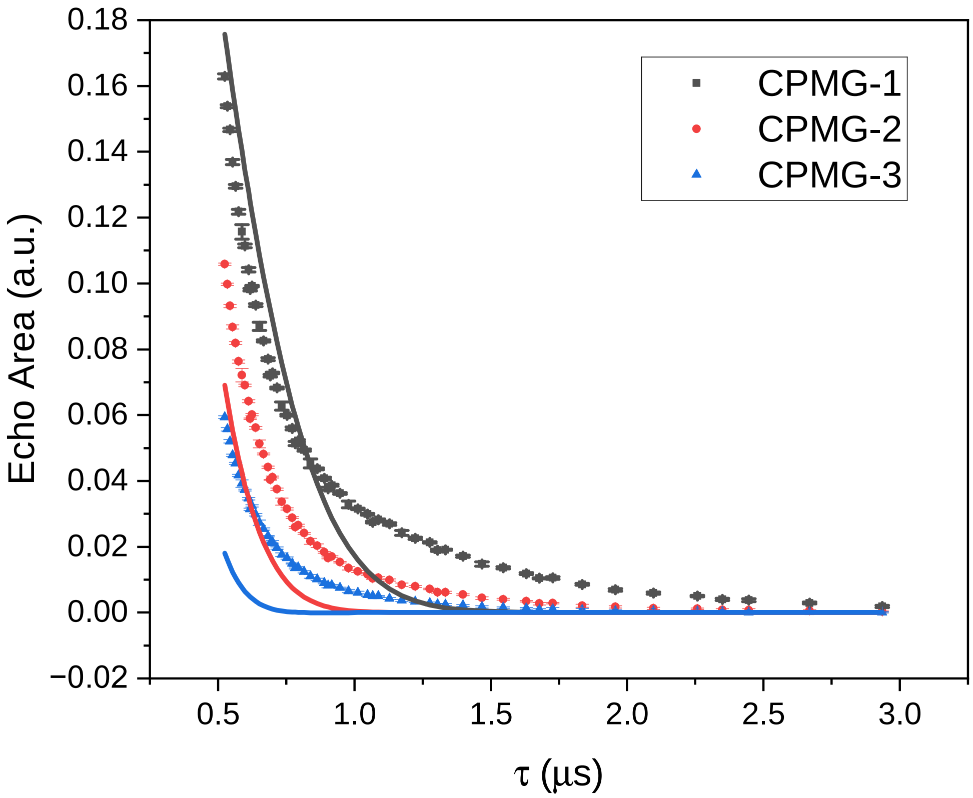

FIG. S10. The result of fitting the zero-field data after forcing $S_E = 0$. The fit is substantially worse in comparison to when S_E is included in the model, cf. Fig. 10(a) in the main text.

linearly with n_p, which we treat as an additional fitting parameter. Figure S12(g-l) show the results of an attempt to employ this model. We indeed see that the additional low-τ peak is no longer visible in the damping-free simulations. However, the fit still fails to adequately reproduce the experimental data, again showing a short T_2 to account for the initial fast decay of the signal. Again, the fundamental issue here appears to be that longer values of T_2, while allowing the simulations to exhibit oscillations, produce signal sizes that are much too large to match the experimental data.

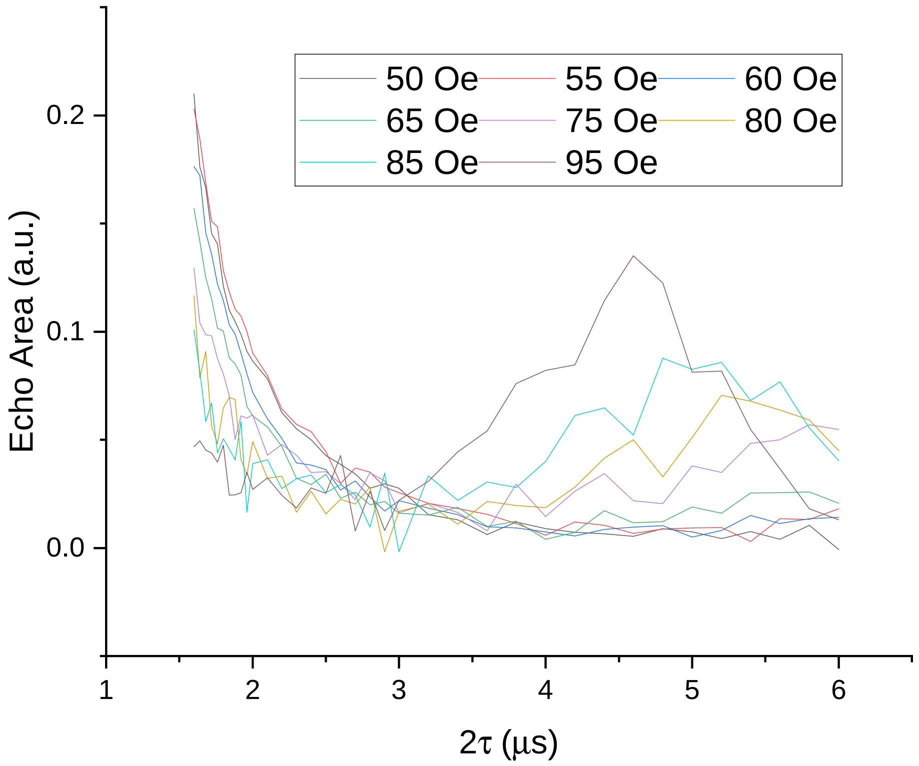

FIG. S11. Hahn echo area at intermediate fields, 50 – 95 Oe. The signal appears to revive after seemingly fully decohering. Data was taken with 5% dilution **2** toluene solution at 1.9 K and 4639 MHz.

---

[1] Excluding $\mathcal{S}_{B_I}$ since the simulations have no dependence on it at zero field.

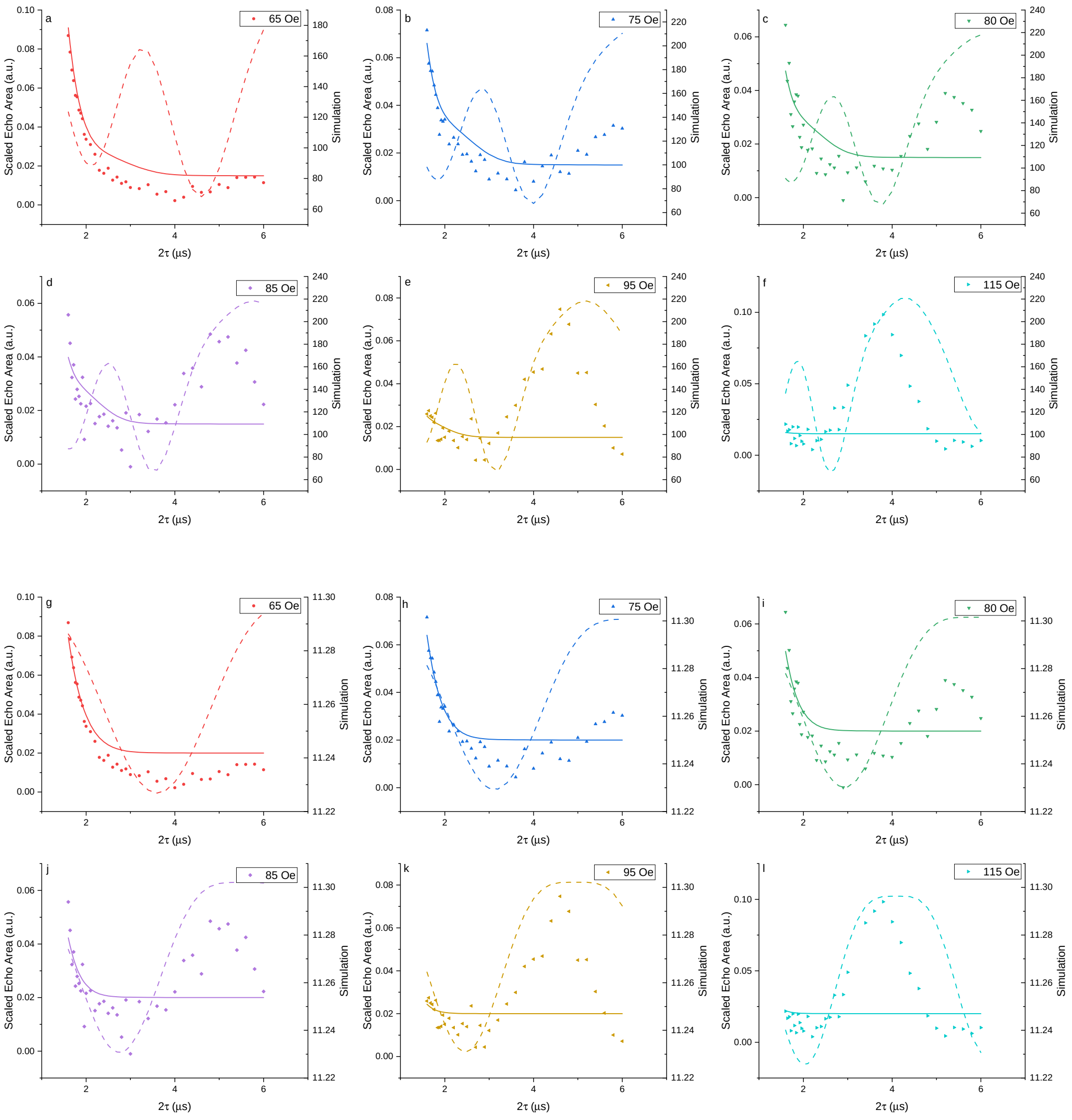

FIG. S12. Attempted fitting at intermediate field values with hyperfine coupling. Panels a–f show the results (solid line) from the same procedure used for Fig. 9, as described in main text, except allowing for refitting the global scaling parameter and offset. Note that with these hyperfine coupling parameters, the undecohered waveform (dashed line) shows additional structure with small τ not present in the data. Panels g–l are the results from fitting using a model of coupling to multiple (n_p) protons with small values of hyperfine coupling, Eq. S21. The modulation frequency is largely the nuclear Larmor frequency as a result.
